# Angelicin improves osteoporosis in ovariectomized rats by reducing ROS production in osteoclasts through regulation of the KAT6A/Nrf2 signalling pathway

**DOI:** 10.1186/s13020-024-00961-7

**Published:** 2024-07-02

**Authors:** Xiao-feng Liu, Yi-tao Liao, Jia-hao Shao, Dan-dan He, Zhi-hong Fan, Ye-Nan Xu, Chao Li, Xian Zhang

**Affiliations:** 1 Nanjing University of Chinese Medicine, Nanjing, 210023 China; 2 Department of Spine, Wuxi Affiliated Hospital of Nanjing University of Chinese Medicine, Wuxi, 214071 China

**Keywords:** Angelicin, Osteoclast, Osteoporosis, Oxidative stress, KAT6A, Nrf2

## Abstract

**Background:**

Angelicin, which is found in Psoralea, can help prevent osteoporosis by stopping osteoclast formation, although the precise mechanism remains unclear.

**Methods:**

We evaluated the effect of angelicin on the oxidative stress level of osteoclasts using ovariectomized osteoporosis model rats and RAW264.7 cells. Changes in the bone mass of the femur were investigated using H&E staining and micro-CT. ROS content was investigated by DHE fluorescence labelling. Osteoclast-related genes and proteins were examined for expression using Western blotting, immunohistochemistry, tartrate-resistant acid phosphatase staining, and real-time quantitative PCR. The influence of angelicin on osteoclast development was also evaluated using the MTT assay, double luciferin assay, chromatin immunoprecipitation, immunoprecipitation and KAT6A siRNA transfection.

**Results:**

Rats treated with angelicin had considerably higher bone mineral density and fewer osteoclasts. Angelicin prevented RAW264.7 cells from differentiating into osteoclasts in vitro when stimulated by RANKL. Experiments revealed reduced ROS levels and significantly upregulated intracellular KAT6A, HO-1, and Nrf2 following angelicin treatment. The expression of genes unique to osteoclasts, such as MMP9 and NFATc1, was also downregulated. Finally, KAT6A siRNA transfection increased intracellular ROS levels while decreasing KAT6A, Nrf2, and HO-1 protein expression in osteoclasts. However, in the absence of KAT6A siRNA transfection, angelicin greatly counteracted this effect in osteoclasts.

**Conclusions:**

Angelicin increased the expression of KAT6A. This enhanced KAT6A expression helps to activate the Nrf2/HO-1 antioxidant stress system and decrease ROS levels in osteoclasts, thus inhibiting oxidative stress levels and osteoclast formation.

**Supplementary Information:**

The online version contains supplementary material available at 10.1186/s13020-024-00961-7.

## Introduction

Low bone mass and the degradation of the microstructure of bone tissue are hallmarks of the condition known as osteoporosis, which is thought to be an inevitable result of aging. Osteoporosis is a worldwide public health issue impacting over 200 million people [1]. Studies have shown that the average peak bone mass occurs significantly earlier in women than in men and that oestrogen levels in the body are the main reason why women are more likely to develop osteoporosis [2]. Especially in postmenopausal women, the decline in oestrogen levels can lead to osteoclast overactivation, causing osteoporosis [3]. Therefore, inhibition of osteoclast generation and differentiation is a primary strategy for treating osteoporosis.

Reactive ROS and antioxidant levels out of balance are the primary cause of oxidative stress [4]. When intracellular ROS production levels are too high, biological macromolecules, such as carbohydrates, lipids, nucleic acids, and proteins, are damaged [5–7]. Studies have proven that high amounts of ROS can cause osteoblast apoptosis and promote osteoclast formation, which leads to bone loss and speeds up the process of osteoporosis [8, 9]. When oxidative stress occurs, the Nrf2- Keap1 complex in the cytoplasm is disrupted, and Nrf2 binds to antioxidant reaction elements (AREs), thereby facilitating the transcriptional activation of the downstream antioxidant gene HO-1 [10, 11]. CO and bilirubin, which are products of HO-1, suppress the ROS/inhibitory nuclear factor-B (NF-B) signalling pathway. This ends nuclear factor-B receptor activator ligand (RANKL) and causes the formation of osteoclasts [12, 13]. Histone acetyltransferases (HATs), which include members of the MYST family, are known to be able to control the Nrf2/ARE/HO-1 feedback mechanism [14, 15]. A related protein, namely, lysine acetyltransferase 6B (KAT6B, also known as MYST4 or monocytic leukaemia zinc finger protein-associated factor (MORF)), belongs to the MYST family and has been reported to promote Nrf2 nuclear retention [16]. According to a recent study, the homolog of KAT6B, KAT6A, also referred to as MYST3 or monocytic leukaemia zinc finger protein (MOZ), uses the Nrf2/ARE/HO-1 signalling pathway to remove reactive ROS that build up in aged mesenchymal stem cells derived from bone marrow (OBMSCs), thus promoting osteogenic differentiation. In the same way, blocking the Nrf2/ARE signalling pathway reverses the gains in OBMSC osteogenesis caused by KAT6A [17, 18].

Angelicin is the main active ingredient of Psoralea, which is a traditional Chinese medicine. Angelicin is a coumarin and has oestrogen-like activity [19]. According to earlier research, angelicin controls the quantity of osteoblasts and osteoclasts by triggering the NF-κB and transforming growth factor (TGF)-β1 signalling pathways [20, 21]. Recent studies have shown that angelicin alleviates ROS overproduction-induced osteoblast damage by inhibiting the levels of oxidative stress in osteoblasts [22, 23]. This implies that osteoporosis can be relieved and prevented by angelicin. Nevertheless, it is still unknown how precisely angelicin controls oxidative stress levels in cells and prevents osteoporosis. Based on our studies, angelicin greatly reduces osteoporosis caused by OVX by inhibiting oxidative stress and osteoclastogenesis in bone tissues. We also discovered that angelicin dose-dependently increased KAT6A expression and that this increase in turn helped activate the Nrf2/HO-1 antioxidant stress system and ameliorate bone mass loss in oophorectomy-induced osteoporosis model rats by decreasing intracellular ROS levels.

## Methods

### Animals

**Fig. 1:**
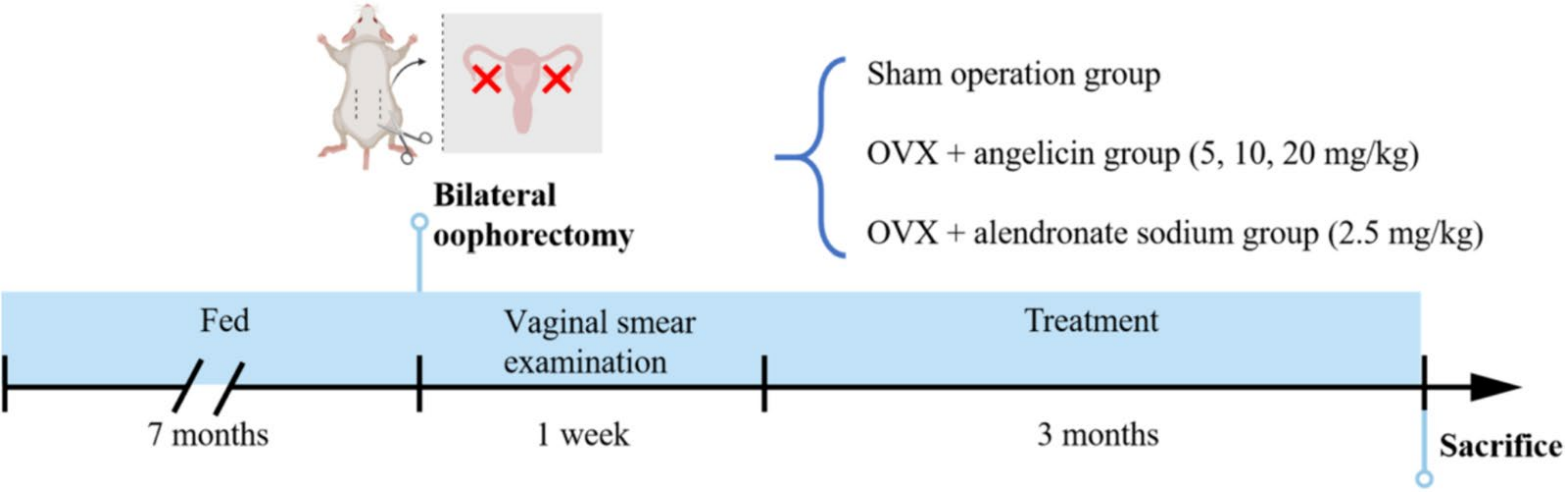
Flow chart of OVX female rat model and drug intervention. Female SD rats were fed to 7 months, underwent bilateral oophorectomy, and were observed for 1 week. After that, the rats in each group were given equal volume of normal saline, angelicin and alendronate sodium orally, once a day, for 3 months (Created with BioRender.com)

Beijing Vital River Laboratory Animal Technology Co., Ltd. supplied the SD rats used in this study. The Ethics Committee of Wuxi Hospital of Traditional Chinese Medicine (SZYYGLJ2021092402) gave its blessing to all of the rat tests. After female SD rats had been fed for 7 months, 30 female rats were randomly selected to undergo bilateral oophorectomy. On the second day after the operation, a vaginal smear examination was performed and repeated once a day for 1 week. The oophorectomy was considered successful if the rats no longer exhibited regular oestrous changes. Six rats were randomly selected to undergo surgical exposure of the ovaries without ovariectomy, and these rats formed the sham operation group [24]. OVX plus angelicin [5 mg/kg, 10 mg/kg, 20 mg/kg; A6780, Solarbio Science & Technology Co., Ltd., (Beijing, China)], OVX plus alendronate sodium [2.5 mg/kg; IA0370, Solarbio Science & Technology Co., Ltd., (Beijing, China)]. The rats in each group were administered equal volumes of normal saline, angelicin or alendronate sodium once a day for 3 months. We then examined the safety of the Angelicin intervention in rats (Supplementary material). The tissue was collected from the right femur of the female SD rat. The samples for Western blotting were frozen in liquid nitrogen and stored at – 80 ℃ for later use. Samples for histochemical staining and immunofluorescence staining were stored and fixed in 4% paraformaldehyde. The process of OVX female rat model and drug intervention is shown in Fig. 1.

### Cell culture and cell viability

RAW264.7 cells [CL-0190, Primitive Cell Life Technology Co., Ltd, (Wuhan, China)] were purchased and added to a medium of 10% foetal bovine serum, 1% penicillin and streptomycin [25]. RAW264.7 cells (1 × 10^3^) were plated on 96-well plates, angelicin and RANKL were added to the previously indicated orifice plate after a day [25]. Following a three-day period of culture, the differentiation of osteoclasts was assessed through TRAP staining. Subsequently, the MTT test was employed to quantify the proliferation of osteoclasts.

### Micro-CT analysis

Each rat's right femur was scanned using Quantum GX μCT imaging equipment (Perkin Elmer Medical Diagnostics Shanghai Co., LTD., Hopkinton, MA, USA) to produce μCT images. After scanning 520 sections (10 μm) from the growth plate at the distal end of the femur, 10 consecutive sections were chosen for study when the condyle on both sides vanished at a distance of 0.1 mm from the growth plate [26, 27]. All the bone trabeculae were separated from each selected section for 2D reconstruction. Trabecular bone microstructure analysis was conducted to obtain the values of the following markers: bone mineral density (BMD), bone volume/tissue volume (BV/TV), trabecular thickness (Tb.Th), trabecular number (Tb.N), and trabecular separation (Tb.Sp).

### Histological staining of bone tissues

The rat femur was immobilized in paraformaldehyde for 24 h, decalcified in 20% EDTA for two weeks to make the bones soft, then decalcified, paraffin-embedded, and sliced 4 weeks later with a measured thickness of 6 μm per femur for eosin (H&E) and tartrate antacid phosphatase (TRAP) staining. They were scanned and photographed with an optical microscope (Olympus BX53, Olympus, Japan) [28]. The cell medium was washed with phosphate buffer solution, it was fixed with paraformaldehyde for 15 min, and the TRAP working liquid was added to the water bath for 60 min, the nucleus was stained with hematoxylin for 10 min, and the cells were washed twice with PBS [29]. In this study, TRAP dyeing kit (catalog number D023-1-1) produced by Nanjing Jiancheng Bioengineering Institute was used. In each sample, five fields of view were randomly selected for a TRAP-positive cell count under an optical microscope.

### ELISA

The blood of rats was obtained through abdominal aorta sampling, and the serum was isolated by centrifugation at 3000 r/min for 15 min at 4 ℃. Serum levels of glutathione (GSH), glutathione peroxidase (GSH-PX), superoxide dismutase (SOD) and catalase (CAT) were detected by ELISA kit. The supernatant of RAW264.7 cells treated with Angelicin was centrifuged to obtain cell fragments, and the levels of GSH, GSH-PX, SOD and CAT in the supernatant were detected according to the manufacturer's instructions. All processes are carried out in accordance with manufacturer's instructions.

### Bone tissue immunohistochemistry (IHC) and immunofluorescence staining

The longitudinal section of the femur was transferred onto a slide and dried at room temperature, and it was incubated overnight at 4 °C with NFATc1 (Abcam; ab2796; 1: 1000), MMP9 (Abcam; AB283575; 1:5000) and HO-1 (CST; 86806; 1:1000) antibodies. The slices were incubated with secondary antibodies, and 3'-diaminobenzidine (DAB) was used to colour them. Every slide was examined and captured on a camera using a microscope (TS2-S-SM, Nikon, Japan). The dried sections of the femur were subjected to a sealing process using 10% goat blood at ambient temperature for 30 min, and were incubated overnight at 4 °C with Nrf2 (CST; 12721; 1:1000) antibodies. Sections were treated with primary and secondary antibodies and were stained by DAPI. The specimens were viewed using a Leica DMi8 microscope manufactured by Carl Zeiss MicroImaging GHBH in Jena, Germany.

### Western blotting

We extracted total protein from the right femoral tissue of rats and RAW264.7 cells in accordance with the directions of the Total Protein Extraction Kit (Signal Antibody LLC, Maryland, USA). PVDF membranes were used to hold the proteins that had been isolated by SDS‒PAGE. Next, the PVDF membranes were incubated overnight at 4 °C with the primary antibodies against β-actin (CST; 4970; 1:1000), KAT6A (ABclonal; a15006; 1:1000), H3 (ABclonal; a2348; 1:1000), H3K9 (ABclonal; a7255; 1:1000), H3K14 (ABclonal; a7254; 1:1000), HO-1 (CST; 86806; 1:1000), Nrf2 (CST; 12721; 1:1000), NFATc1 (Abcam; ab2796; 1: 1000), Lamin B (CST; 17416; 1:1000), p-NF-κB (CST; 3033; 1:1000), NF-κB (Abcam; ab32536; 1:1000) and c-Fos (Abcam; ab222699; 1:1000), after which the corresponding secondary antibodies were applied for 1 h at room temperature. Finally, immuno-luminescent bands were acquired by chemiluminescence imaging tools (Alliance Q9 Advanced, Uvitec Ltd., Cambridge, UK).

**Table 1:** Primers used in this study

Primer	Sequences (5ʹ-3ʹ)
RAT-NFATc1-F	GGGCATCTCAGCTGTTCCTT
RAT-NFATc1-R	GCAGGGTTGCTGTAGATGGT
RAT-Ctsk-F	CCGTGGTGAGCTTTGCTCTA
RAT-Ctsk-R	AGGTGATTCATGGCCAGCTC
RAT-Oscar-F	GCTGTCTACTCTCTGTGAGCTG
RAT-Oscar-R	AGAGTCCAAATCCCCAGGCA
RAT-Trap-F	ATACTGTCTGCCAACCCTGC
RAT-Trap-R	TGGTTTCTTGACCACCTTTTTGA
RAT-HO-1-F	TGCACATCCGTGCAGAGAAT
RAT-HO-1-R	AGGGAAGTAGAGTGGGGCAT
MUS-NFATc1-F	CCAGCTTTCCAGTCCCTTCC
MUS-NFATc1-R	AGGTGACACTAGGGGACACA
MUS-Ctsk-F	CTCCAGTCAAGAACCAGGGC
MUS-Ctsk-R	GGTCATATAGCCGCCTCCAC
MUS-Oscar-F	ACTCCAGCTGTCGACTCTCT
MUS-Oscar-R	TTTGAAGAGTGCAAACCGCC
MUS-Trap-F	TTGTTGACAGCGGTCCATCT
MUS-Trap-R	GGTGCCCTCCTTCTTAACCC
MUS-HO-1-F	GGAAATCATCCCTTGCACGC
MUS-HO-1-R	TGTTTGAACTTGGTGGGGCT

### Quantitative RT-PCR assay

For RAW264.7 cells and bone tissue, total RNA was extracted using TRIzol reagent (Invitrogen, Carlsbad, CA, USA). cDNA was prepared with a cDNA reverse transcription kit (Applied Biosystems, Thermo Fisher Scientific, Inc.). SYBR Green PCR Master Mix (Applied Biosystems) was then used to conduct RT-PCR 9 (Table 1).

### Pit staining of bone slices

Bovine femur sections were selected for the osteoclast bone resorption test. The thickness of the section was 0.4 mm, the diameter was 6 mm, and the section was suitable for 96-well plates. RAW264.7 cells were plated on bone sections at a density of 1 × 103 per well. Six hours later, 100 ng/ml RANKL was added, and different concentrations of angelicin were added. The medium was replaced with fresh medium every 2 days. After continuous culture for 8 days, surface changes in the bone slices were observed under an optical microscope every day until pits and grooves on the osteoclasts were visible after bone absorption. Statistical analyses were performed by taking images under an optical microscope.

### ROS level measurement

To examine the generation of ROS, frozen femur tissue slices and RAW264.7 cells were stained with a DHE reaction combination (Beyotime; Shanghai, China) [30]. As mentioned above, an ROS detection kit was used to measure ROS production. Flow cytometry (FACSCalibur flow cytometry; BD Biosciences) to detect fluorescence intensity.

### Transfection test and luciferase test

Two × 10^5^ RAW264.7 cells were seeded in 12-well plates. KAT6A siRNA (Ribobio, Guangzhou, China) was transfected into RAW264.7 cells in the experimental group Following the manufacturer's recommendations, Lipofectamine 2000 (Invitrogen, USA) was utilized as the transfection reagent. For RNA expression analysis, RAW264.7 cells were harvested 48 h in advance, and for protein expression analysis, they were harvested 72 h later. The cells underwent a 24-h treatment with or without angelicin after being co-transfected with pNrf2-TA-luc (Sangon Biotech Co., Ltd., Shanghai, China) for 24 h. The Dual-Luciferase Reporter kit (Promega, Wisconsin, USA) was used to measure the luciferase activity of the cell lysate. Promega identified luciferase, and we employed the Dual Luciferase system (dual luciferase reporter gene assay).

### Chromatin immunoprecipitation (ChIP)

The Chipit Express kit (Active Motif, Carlsbad, CA) was used to cultivate RAW264.7 cells on a petri dish with six collagen-coated cells, as per the instructions. Separate petri dishes were filled with DMSO and angelicin. Following a 2 h period, the cells were fixed for ten minutes using 1% formaldehyde. After washing, fixing was discontinued by adding a glycine solution. The cells were washed again to harvest the cells. By using centrifugation and precipitation, the nucleus was extracted. An enzyme-cut mixture was applied to the nuclear extracts for 12 min at 37 °C. The chromatin that had been cut was collected by centrifugation at 4 °C for 10 min after EDTA was applied to halt the enzyme process. Using IgG as the control, the acetylation levels of H3K9 and H3K14 were determined (Cell Signaling Technology; 14708; 1:5000). The precipitated DNA was amplified using real-time PCR.

### Coimmunoprecipitation (CoIP)

The cells were treated with immunoprecipitation (IP) buffer (Beyotime Biotechnology) and cOmpleteTM protease inhibitor cocktail tablets (5 mg/ml; Roche, Basel, Switzerland) for 15 min at 4 °C. The extracts were gathered and put to use. An appropriate cell lysate was mixed with anti-MYC or anti-Flag antibodies, and the mixture was shaken for 12 h. Then, protein A/G and Santa Cruz were added to the cell lysate containing the antibody, and it was incubated for 4 h at 4 °C. Three washings were performed using cooled PBS buffer. Antibodies against Nrf2 (CST #12721) and KAT6A (ABclonal a15006) were used. Subsequently, the sample was separated by SDS-PAGE. WB analysis led to the detection of protein expression.

### Statistical analysis

The analytic software SPSS 25.0 and Graphpad Prism 8.0.1 were utilized to analyze the data. The information is displayed as the mean ± s.d. or mean ± s.e.m. To determine if the measurement data fit the normal distribution, SPSS 25.0 was utilized. The one-way analysis of variance (ANOVA) function of Graphpad Prism 8.0 was used to examine group differences if the data fit the normal distribution. Tukey's multiple comparison tests were then performed for further analysis. When data does not follow a normal distribution, the Kruskal–Wallis test is performed. A statistically significant difference was considered as P < 0.05 in all comparisons.

## Results

### Angelicin protects against bone mass loss in OVX model rats

**Fig. 2:**
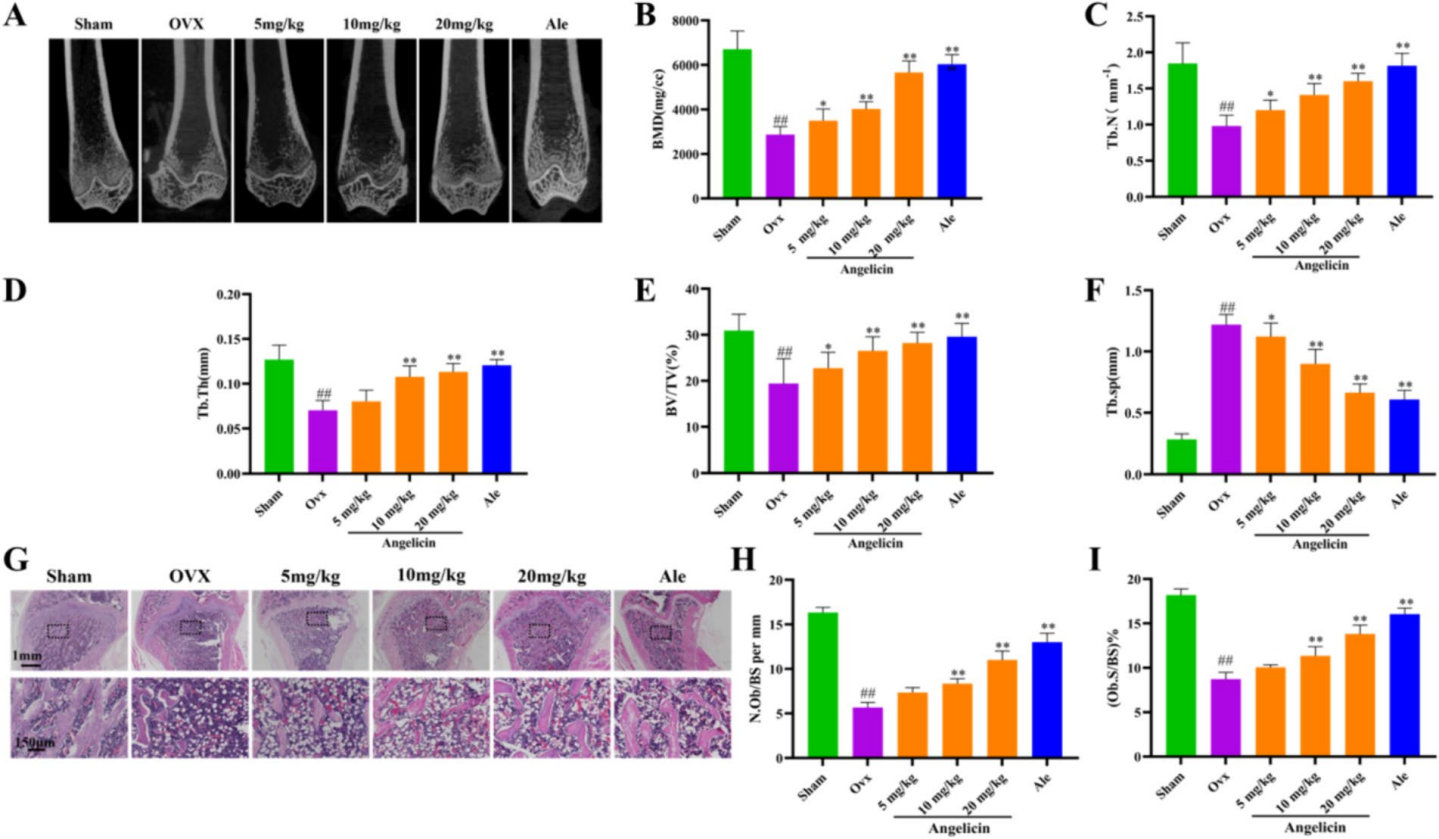
Angelicin protects against bone mass loss in OVX model rats. **A** Micro-CT image of the end of a rat femur. **B**–**F** Indicators were analyzed (n = 6). *P < 0.05, **P < 0.01. **G** H&E staining of femur tissue sections. **H**, **I** N.Ob/BS and Ob.S/BS on each bone surface

To evaluate the effects of angelicin on the structural characteristics of bones in rats, we performed μCT analysis of distal femur metaphyseal trabecular bones. The results revealed that the Tb. Sp values increased and the BMD, Tb. N, Tb. Th, and BV/TV values decreased in the OVX group; however, these effects were dramatically reversed in the OVX + angelicin group (Fig. 2A–F). H&E staining of femur tissue sections from the OVX, OVX + angelicin, and sham operation groups also verified that angelicin decreased bone mass loss in OVX model rats (Fig. 2G). Moreover, we determined the quantity of osteoblasts (N.Ob/BS) and osteoblast surfaces (Ob.S/BS) for each surface of the bone. The findings indicated that the administration of angelicin considerably raised N.Ob/BS and Ob.S/BS in comparison to the OVX group (Fig. 2H, I). These findings imply that in rats with OVX-induced osteoporosis, angelicin prevents the loss of bone mass.

### Angelicin reduces osteoclast numbers and osteoclast differentiation in bone tissues of OVX-induced osteoporosis model rats

**Fig. 3:**
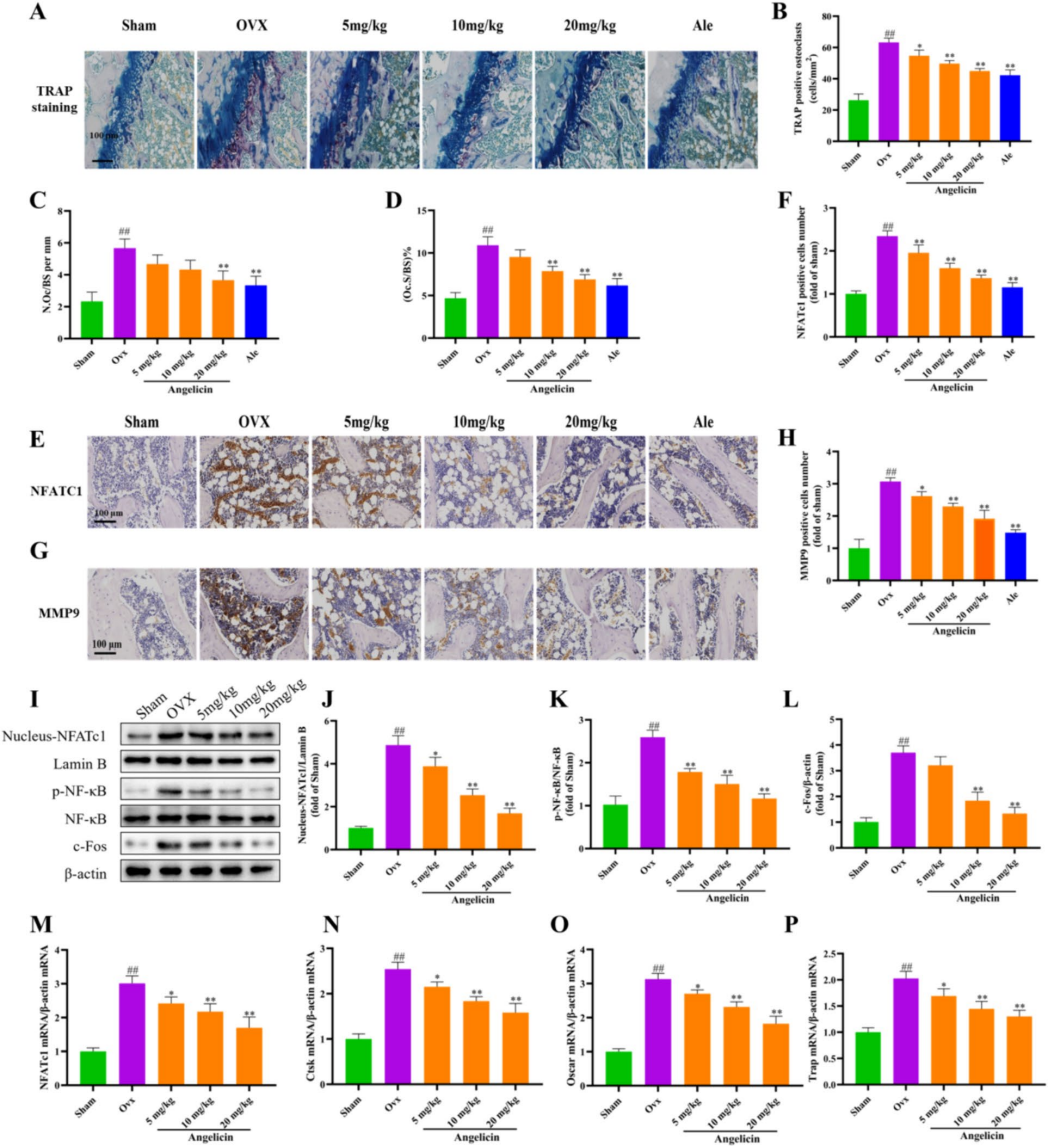
Angelicin can reduce osteoclast formation and inhibit osteoclast-related gene expression. **A** Rats in the sham surgery, OVX, OVX + angelicin, and positive control groups had bone tissue slices that were stained with TRAP, as seen in the representative photos. Scale bar = 100 μm. TRAP-positive cells in each field. **B** Quantitative evaluation of the TRAP-positive cell count (n = 3). **C**, **D** N.Oc/BS and Oc.S/BS on each bone surface. **E**, **G** Representative images showing IHC staining of femoral bone sections from sham surgery, OVX, and OVX + angelicin rats. **F**, **H** NFATc1- and MMP9-positive cells (n = 3). **I** WB images showing significantly downregulated nuclear expression of NFATc1, Lamin B, p-NF-κB, NF-κB, and c-Fos in the sham operation, OVX and OVX + angelicin groups. **J**–**L** Expression levels of NFATc1, p-NF-κB, and c-Fos (n = 3). **M**–**P** Expression levels of NFATc1 mRNA, Ctsk mRNA, Oscar mRNA and Trap mRNA were quantitatively analyzed by qRT-PCR (n = 3). *P < 0.05, **P < 0.01

The histology of the femur was examined in rats. TRAP staining showed that compared with the OVX group, angelicin inhibited osteoclast formation (Fig. 3A and B). When comparing the OVX group to the osteoclast group, we found that the osteoclast surface count (Oc.S/BS) and number of osteoclasts (N.Oc/BS) per bone surface were significantly lower with the angelicin intervention (Fig. 3C and D). The differentiation of osteoclasts is related to the expression level of specific genes of osteoclasts [31, 32]. The expression of the MMP9 and NFATc1 genes in osteoclasts was assessed by IHC. When comparing the OVX group to the sham surgery group, there was a notable increase in the expression of MMP9 and NFATc1. However, in contrast to the OVX group, angelicin decreased NFATc1 and MMP9 levels (Fig. 3E–H). Next, we measured the expression of proteins that play crucial roles during osteoclast differentiation. In response to angelicin treatment, the protein expression levels of the osteoclast-related nuclear transcription factors NFATc1, p-NF-κB, NF-κB, and c-Fos were significantly reduced (Fig. 3I–L). Quantitative real-time PCR analysis confirmed that angelicin significantly downregulated the mRNA expression of NFATc1 and its downstream target genes (Ctsk, Oscar, and Trap) in osteoclasts from femur tissues (Fig. 3M–P). Overall, these findings show that by reducing both the quantity of osteoclasts and the expression of genes linked to osteoclast development, angelicin decreases the generation of osteoclasts in rats with OVX-induced osteoporosis.

### Angelicin inhibited ROS production and activated the Nrf2 signalling pathway in osteoclasts of femur tissues

**Fig. 4:**
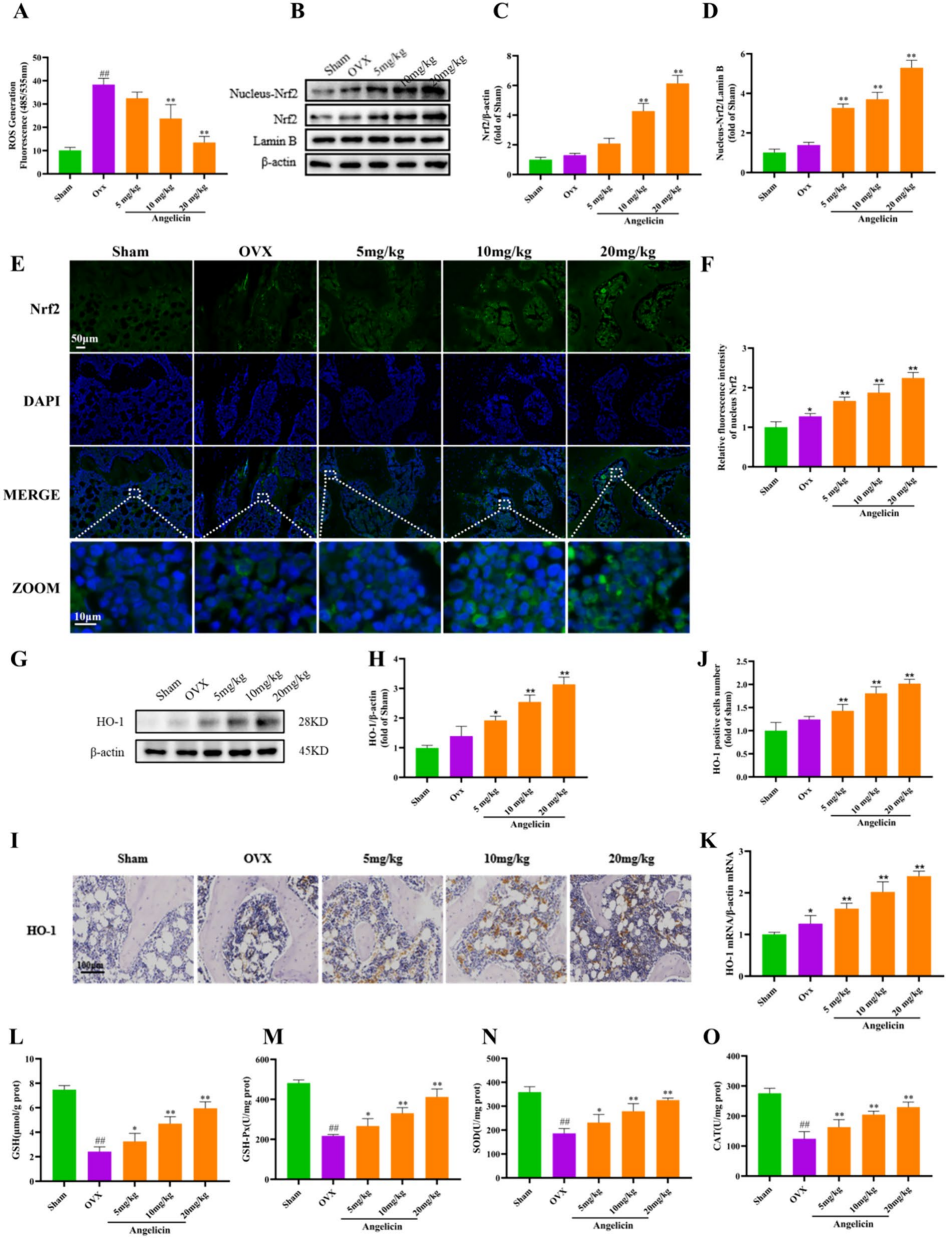
Activation of the Nrf2 antioxidant system by angelicin reduces ROS production in the osteoclasts of femur tissues. **A** Quantitative analysis of ROS levels in rat osteoclasts from the sham operation, OVX and OVX + angelicin groups, as shown by DHE fluorescence staining (n = 3). **B** WB images were used to determine that the rat osteoclasts expressed the Nrf2 protein. **C**, **D** Analysis of Nrf2 protein expression levels (n = 3). **E** Illustrations of representative immunofluorescence pictures demonstrating Nrf2 expression (together with DAPI) in the osteoclasts of rats from the OVX and OVX + angelicin groups. Scale = 50 µm. **F** Quantitative analysis of Nrf2 (plus DAPI) protein expression (n = 3). **G** WB examining HO-1 protein expression in osteoclasts from the sham operation, OVX and OVX + angelicin groups. **H** HO-1 protein expression levels (n = 3). **I**, **J** HO-1 protein levels were measured by IHC. Scale = 100 µm. (n = 3). **K** The mRNA expression of Nrf2 in the osteoclasts of the rat’s right femur. (n = 3). **L**–**O** The levels of serum GSH, GSH-PX, SOD and CAT were measured by ELISA. *P < 0.05, **P < 0.01

ROS are intracellular messenger molecules that are essential for initiating downstream signalling pathways at the onset of osteoclast oogenesis [33, 34]. Some studies showed that angelicin could affect osteoclast differentiation by inhibiting the levels of intracellular ROS [35]. We used DHE to measure ROS levels. The findings demonstrated that the bone tissue ROS levels in the OVX group were much higher, whereas the OVX + angelicin group showed the opposite result (Fig. 4A). Cells have a variety of antioxidant protective mechanisms, and the more well-known regulatory mechanism involves the protective enzyme Nrf2 [36]. Next, we used WB and immunofluorescence to quantify Nrf2 protein expression in osteoclasts. The findings revealed that, in a dose-dependent manner, angelicin enhanced intracellular Nrf2 protein expression in comparison to the OVX group (Fig. 4B–F). One of Nrf2's downstream factors, HO-1, is essential for lowering intracellular ROS levels [11, 37]. According to WB and IHC tests, rats in the OVX group had lower levels of HO-1 protein in bone tissues, whereas rats in the OVX + angelicin group had higher levels of HO-1 protein in bone tissues (Fig. 4G–J). Moreover, quantitative RT-PCR confirmed that HO-1 gene expression was upregulated in the bone tissue cells of rats in the OVX + angelicin group (Fig. 4K). Furthermore, GSH, GSH-PX, SOD, and CAT are crucial components of the organism's antioxidant system because they transform related reactive oxygen species into innocuous compounds, shielding cells from injury [22]. As a result, we used ELISA to assess the effects of angelicin on the levels of GSH, GSH-PX, SOD, and CAT in rats. The findings demonstrated that, in contrast to the OVX group, angelicin produced dose-dependent increases in the levels of GSH, GSH-PX, SOD, and CAT (Fig. 4L–O). These data suggest that angelicin inhibits ROS production in the osteoclasts of rats with OVX-induced osteoporosis by activating the Nrf2/HO-1 pathway.

### Angelicin activates the Nrf2 antioxidant system through KAT6A

**Fig. 5:**
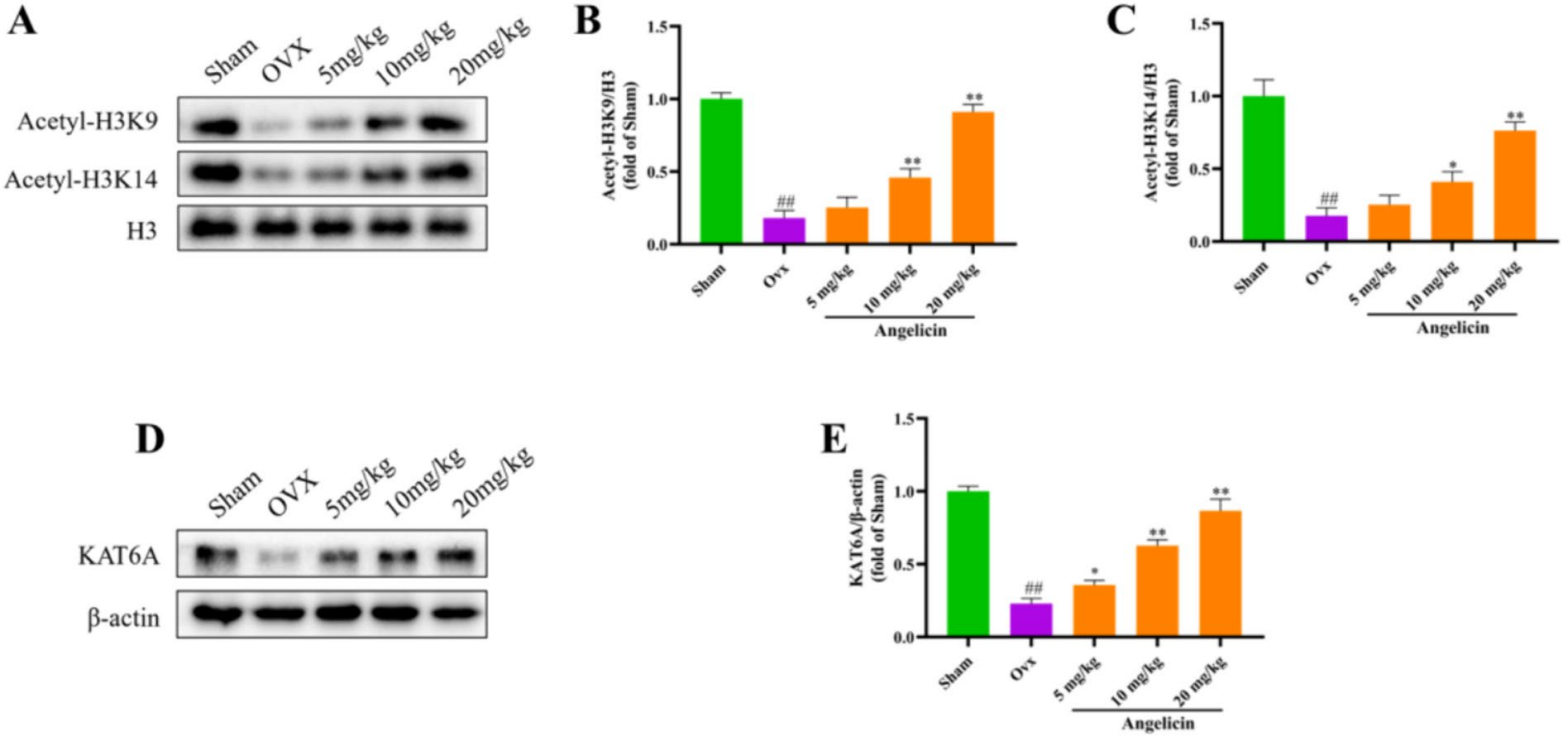
Angelicin activates the Nrf2 antioxidant system through KAT6A. **A** Representative WB images showing histone H3K9 and H3K14 acetylation levels in the sham operation, OVX and OVX + angelicin groups. **B**, **C** The acetylation levels of histone H3K9 and H3K14 were quantitatively analyzed (n = 3). **D** Rat KAT6A expression levels as seen in WB images. **E** The expression of KAT6A was quantitatively analyzed (n = 3). *P < 0.05, **P < 0.01

The levels of histone acetylation in the nucleus of femur tissues from the sham operation, OVX, OVX + angelicin, and positive control groups were then measured using WB. The findings demonstrated that acetylation levels of histones H3K9 and H3K14 were greater in OVX + angelicin group rats (Fig. 5A–C). KAT6A is a HAT that is specific for histone H3 acetylation. WB results confirmed that angelicin increased KAT6A protein expression (Fig. 5D and E).

### Angelicin inhibits the differentiation of osteoclasts in vitro

**Fig. 6:**
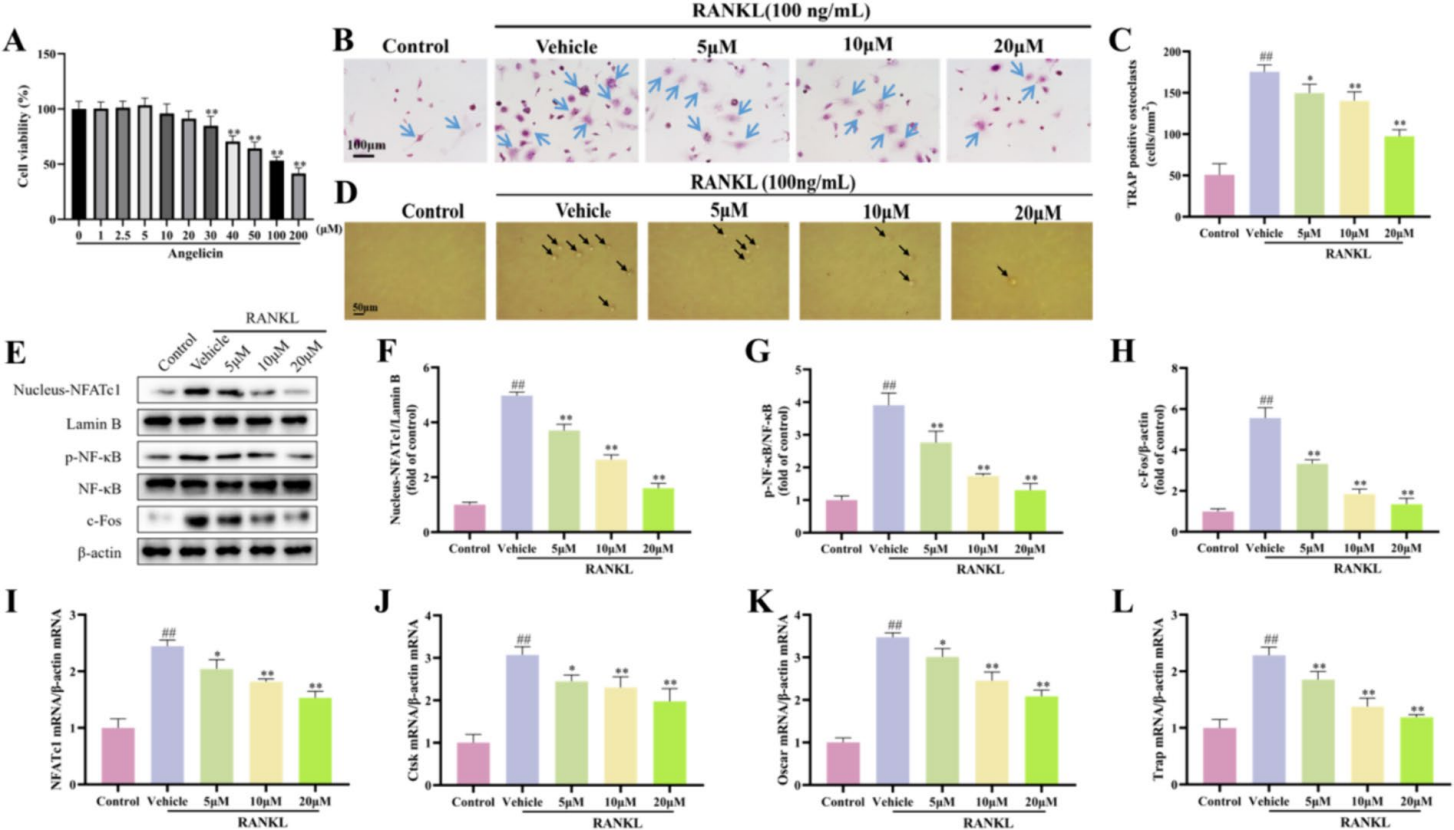
Angelicin inhibits osteoclast differentiation in vitro. **A** The MTT assay was used to quantitatively analyze RAW264.7 cell viability after treatment with 0, 1, 2.5, 5, 10, 20, 30, 40, 50, 100, and 200 µM angelicin (n = 6). **B** RAW264.7 cells were treated with angelicin. Trap staining showed the osteoclasts. Scale bar = 100 μm. **C** Counting of Trap-positive cells for analysis (n = 3). **D** Representative image showing pits formed by osteoclast absorption in the bone section. **E**–**H** WB depicting the expression of important nuclear proteins such as NFATc1, Lamin B, p-NF-B, NF-B, and c-Fos. (n = 3). **I**–**L** Expression of NFATc1 mRNA levels and its downstream target genes Ctsk, Oscar, and Trap were quantitatively analyzed by qRT-PCR (n = 3). *P < 0.05, **P < 0.01

In vitro experiments were used to better assess the effect of angelicin on osteoclasts. [25, 38]. The MTT experiment revealed a significant decrease in RAW264.7 cell viability after treatment with 30 µM angelicin. Ultimately, we chose 5, 10, and 20 µM angelicin as the drug doses that were used for subsequent experiments (Fig. 6A). Angelicin was found to inhibit osteoclast development, as determined by TRAP staining, when compared to the RANKL-induced group [39] (Fig. 6B and C). In addition, we tested the phagocytic function of osteoclasts, and the results showed that angelicin intervention could reduce the bone absorption capacity of osteoclasts (Fig. 6D). We measured the expression of proteins that play crucial roles during osteoclast differentiation. The findings demonstrated that RAW264.7 cells, angelicin decreased the protein expression of the associated transcription factors NFATc1, p-NF-κB, NF-κB, and c-Fos (Fig. 6E–H). When macrophages differentiate into osteoclasts, the osteoclast-specific gene NFATc1 is upregulated [40]. Quantitative RT- PCR confirmed the relative mRNA expression of NFATc1 and its downstream target genes Ctsk, Oscar, and Trap was significantly upregulated, but angelicin significantly reduced the expression levels of these transcripts (Fig. 6I–L). These findings suggest that in vitro, angelicin significantly suppresses the expression of genes linked to osteoclast differentiation, preventing the development of osteoclasts.

Angelicin reduces ROS production in osteoclasts by activating the intracellular Nrf2 antioxidant system.

**Fig. 7:**
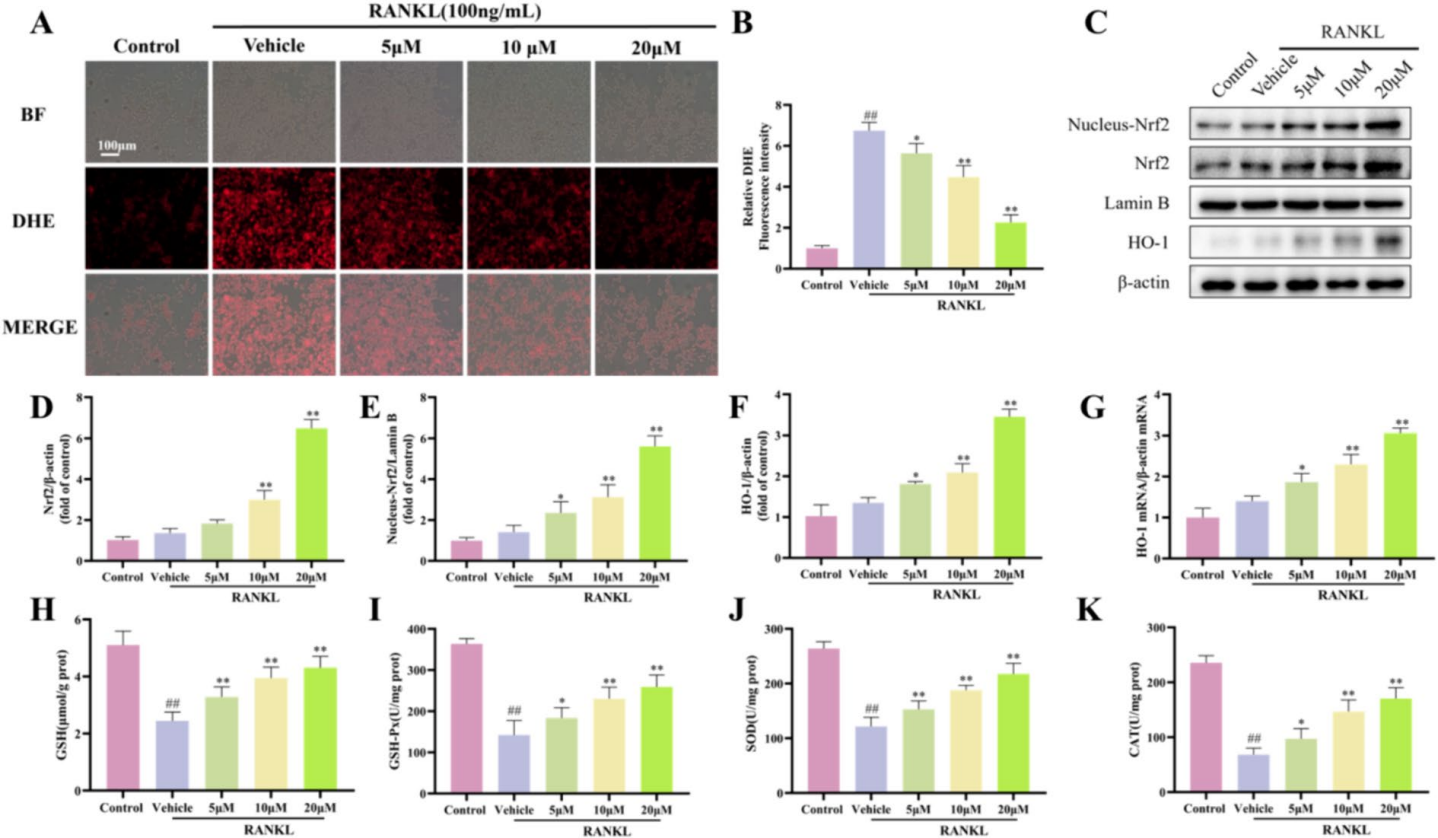
Angelicin reduces ROS production in osteoclasts by activating the intracellular Nrf2 antioxidant system. **A** Representative DHE images showing the ROS content in RAW264.7 cell-derived osteoclasts treated with angelicin. Scale = 100 μm. **B** The ROS levels of DHE-stained osteoclasts were quantitatively analyzed. (n = 3). **C** WB images displaying the expression of HO-1, Lamin B, and Nrf2, three intracellular antioxidant-related proteins. **D**–**F** Nrf2, Lamin B, and HO-1 in osteoclasts were quantitatively analyzed. (n = 3). **G** The expression of the HO-1 antioxidant gene was studied at the mRNA level using quantitative RT-PCR (n = 3). **H**–**K** The levels of GSH, GSH-PX, SOD and CAT in RAW264.7 cells were measured by ELISA. *P < 0.05, **P < 0.01

Next, we used DHE staining to measure ROS levels in osteoclasts. ROS levels were elevated in RANKL-treated RAW264.7 cells but significantly decreased in cells that were also treated with angelicin (Fig. 7A and B). In addition, WB revealed increased expression of antioxidant-related proteins (Nrf2, Lamin B, and HO-1) (Fig. 7C–F). Furthermore, angelicin increased the levels of the antioxidant gene HO-1, which was decreased in RANKL-treated RAW264.7 cells (Fig. 7G). In addition, ELISA assay showed that GSH, GSH-PX, SOD and CAT levels increased in RANKL-induced RAW264.7 cells treated with Angelicin (Fig. 7H–K). These results suggest that angelicin upregulates HO-1 expression by activating the intracellular Nrf2 antioxidant system, thereby reducing ROS production in RANKL-induced osteoclasts.

### Angelicin activates the Nrf2 antioxidant system by enhancing the expression of KAT6A

**Fig. 8:**
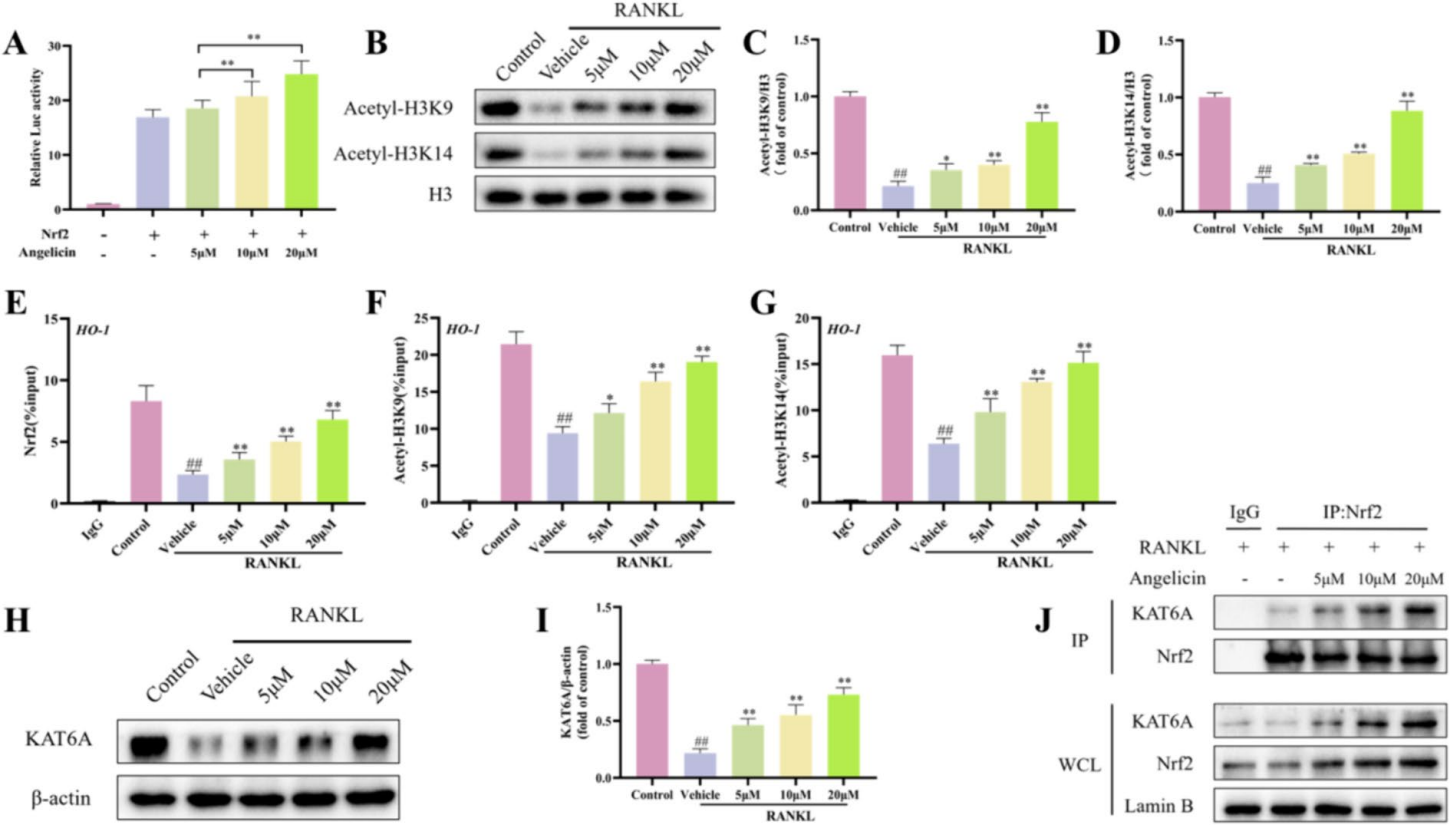
Angelicin activates the Nrf2 antioxidant system through KAT6A. **A** Histograms showing the fluorescent enzyme activity of Nrf2 in osteoclasts after treatment with angelicin. (n = 6). **B** The acetylation levels of histones H3K9 and H3K14 in osteoclasts were demonstrated by representative WB. **C**, **D** The histogram shows that the acetylation levels of histones H3K9 and H3K14 were increased in osteoclasts after treatment with 0, 5, 10, and 20 µM angelicin (n = 3). **E**–**G** Histograms showing the acetylation levels of histones H3K9 and H3K14 near HO-1 in osteoclasts after treatment with angelicin (n = 3). **H** WB results demonstrating the expression of KAT6A in osteoclasts. **I** Histograms showing the expression of KAT6A in osteoclasts after treatment with angelicin. (n = 3). **J** Representative CoIP results showing that angelicin promotes the binding of Nrf2 and KAT6A. *P < 0.05, **P < 0.01

Angelicin strongly stimulated Nrf2 fluorescence enzyme activity (Fig. 8A). H3K9 and H3K14 have been described as targets for KAT6A [41]. WB showed that the H3K9 and H3K14 were decreased in RAW264.7 cells (Fig. 8B–D). ChIP demonstrated that angelicin significantly enhanced the H3K9 and H3K14 near HO-1 (Fig. 8E–G). KAT6A and Nrf2 are upstream molecules of HO-1. WB shows that angelicin increased the expression of KAT6A in RANKL-induced osteoclasts. Moreover, CoIP proved that angelicin promoted the binding of Nrf2 and KAT6A (Fig. 8H–J).

### KAT6A interference inhibits the activation of the Nrf2 antioxidant system by angelicin

**Fig. 9:**
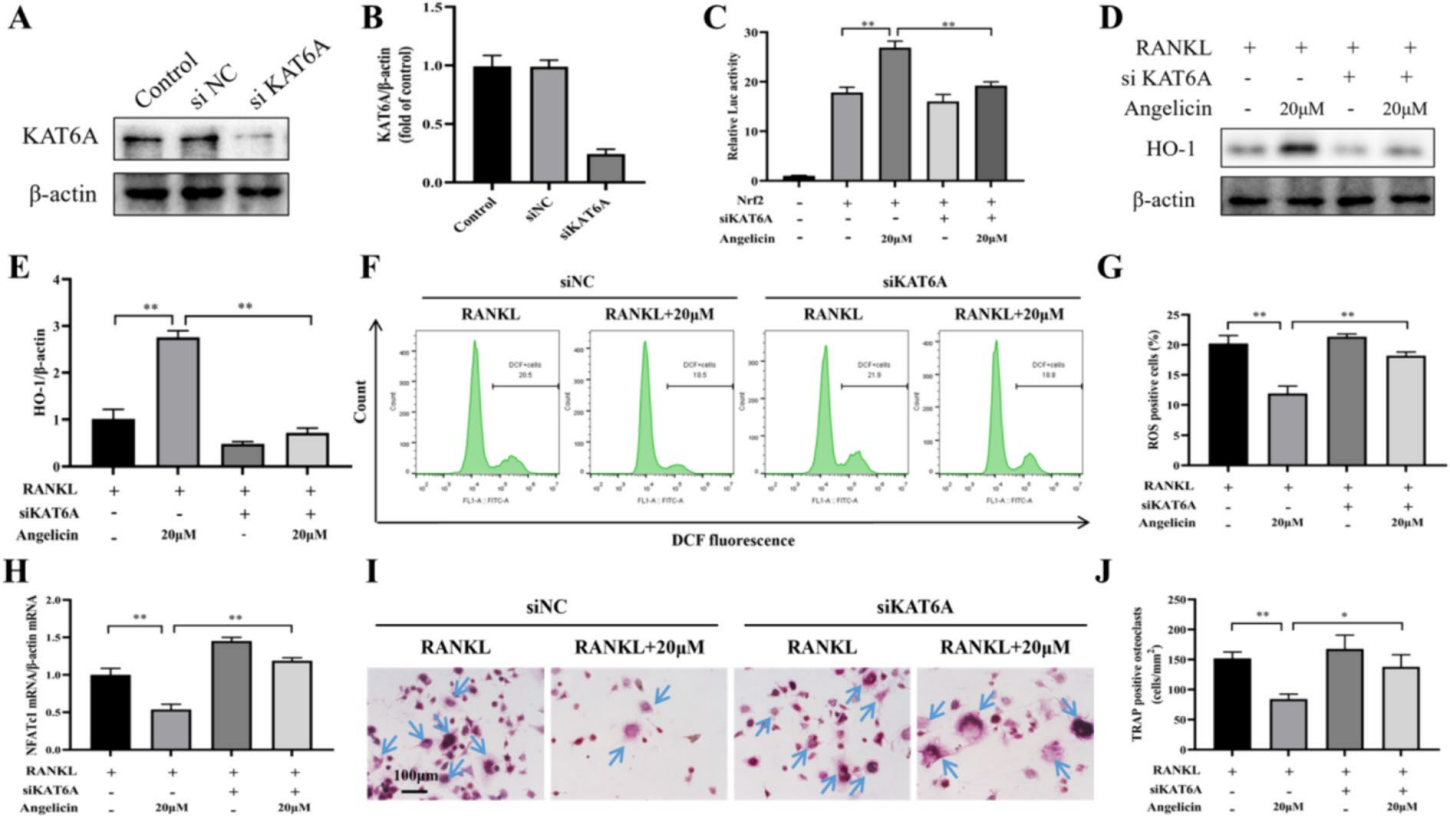
KAT6A interference inhibits the activation of the Nrf2 antioxidant system by angelicin. **A**, **B** WB images demonstrating the KAT6A protein levels in RAW264.7 cells with KAT6A knockout. (n = 3). **C** The relative luciferase activity in RAW264.7 cells treated with KAT6A deletion, Nrf2 and angelicin is displayed in the histogram. (n = 6). **D**, **E** WB images demonstrating the levels of HO-1 in RAW264.7 cells. (n = 3). **F**, **G** Flow cytometry analysis showed the levels of ROS in control and KAT6A-knockout RAW264.7 cells treated with RANKL or RANKL + 20 µM angelicin. (n = 3). **H** Histogram of qRT‒PCR results showing the levels of NFATc1 mRNA in osteoclasts treated with KAT6A deletion, Nrf2 and angelicin (n = 3). **I**, **J** TRAP staining demonstrating the number of osteoclasts (n = 3). *P < 0.05, **P < 0.01

To further support these findings, we successfully constructed a KAT6A protein knockout model, and WB analysis showed that it significantly inhibited KAT6A expression in RAW264.7 cells (Fig. 9A, B). A double luciferin assay showed that 20 µM angelicin increased the relative luciferin activity of Nrf2 in RAW264.7 cells, and siKAT6TA inhibited this increase (Fig. 9C). Next, WB showed that angelicin upregulated HO-1 protein expression in cells, but KAT6A knockout significantly reversed this effect (Fig. 9D, E). Flow cytometry showed that 20 µM angelicin significantly inhibited ROS production in RAW264.7 cells, and KAT6A knockout also reversed this effect (Fig. 9F and G). Finally, we used quantitative RT-PCR and TRAP staining to analyze osteoclast differentiation. In RAW264.7 cells, 20 µM angelicin inhibited the expression of the NFATc1 and reduced osteoclast differentiation, and KAT6A knockout significantly reversed these effects of angelicin on osteoclasts (Fig. 9H–J). These data suggest that angelicin reduces intracellular ROS production and inhibits osteoclast differentiation by activating the osteoclast KAT6A/Nrf2/HO-1 pathway.

## Discussion

As the global population ages, the incidence of osteoporosis is increasing yearly. Long-term medication is needed to increase bone mass in patients with osteoporosis. Currently, most clinical drugs used to treat osteoporosis include calcium and vitamins, oestrogen receptor modulators, bisphosphonates, and so on. However, long-term use of these drugs can result in specific toxic side effects, decreasing their effectiveness [42]. The ability of Chinese herbal plants to treat osteoporosis has gradually been revealed. Research has indicated that the protective effects of natural compounds that are extracted from plants (e.g., isoflavones, puerarin, phytoestrogens, etc.) against diseases such as osteoporosis may allow these extracts to be used as effective alternatives to avoid the side effects of conventional treatments [43]. Previous studies have shown that the active ingredient of *Cullen corylifolium* exerts anti-osteoporosis effects in vitro [21, 44]. In this experiment, angelicin boosted bone density in rat femur tissue substantially. These results suggest that angelicin protects against bone mass loss in rats.

Oxidative stress is mainly caused by the overproduction of ROS, damage to the antioxidant system, or a combination of these two factors [45, 46]. ROS are oxygen-based chemicals that include O2^−^ and OH^−^anions as well as H_2_O_2_ [47]. The accumulation of ROS in bone tissue has been observed in degenerative diseases, such as osteoporosis [46]. Intracellular ROS accumulation inhibits osteoblast differentiation and promotes osteoclast differentiation [48, 49]. Previous studies have shown that angelicin can inhibit oxidative stress in postmenopausal mice with osteoporosis, reverse H_2_O_2_-induced ROS production in osteoblasts, and inhibit osteoblast apoptosis [22, 23]. In this experiment, we observed that angelicin reduced ROS production in osteoclasts and that angelicin downregulated the genes (NFATc1 and MMP9) and key proteins (NFATc1, p-NF-κB, NF-κB, and c-Fos) during osteoclast differentiation.

Epigenetics was first proposed by Conrad Waddington, and epigenetic modifications can regulate the fate of cells during human development [50]. In epigenetics, histone modification is crucial and is believed to be crucial in controlling gene expression [51]. The acetylation of histones mediates various physiological processes in cells. HAT and histone deacetylases (HDACs) determine whether histones undergo acetylation. KAT6A, which is a HAT that is specific for histone H3 acetylation, was first identified in acute myeloid leukaemia in 1996 [52]. KAT6A reduces ROS production in OBMSCs by regulating the Nrf2/ARE antioxidant system [18]. Our study found for the first time that angelicin further activated the Nrf2 antioxidant system by upregulating the level of KAT6A in osteoclasts, increasing the acetylation levels of histone H3K9 and H3K14. Again, KAT6A siRNA had the opposite effect.

However, this study has many shortcomings. Initially, it was shown that angelicin exerts an anti-osteoporosis effect by activating the Nrf2 antioxidant system in osteoclasts. However, the pathogenesis of osteoporosis involves multiple signalling pathways, and a recent network pharmacological and molecular analysis preliminarily verified that angelicin could exert anti-osteoporosis effects through the PI3K/AKT/mTOR pathway [44]. On these fronts, however, the effects of angelicin are unknown. Second, the effect of angelicin on osteoblasts needs further investigation. Third, to assess the pathological mechanism of osteoporosis, it is necessary to consider the function of the immune system and the release of inflammatory substances. Therefore, the effects of Angelicin on the immune system and inflammatory factors warrants attention.

## Conclusion

**Fig. 10:**
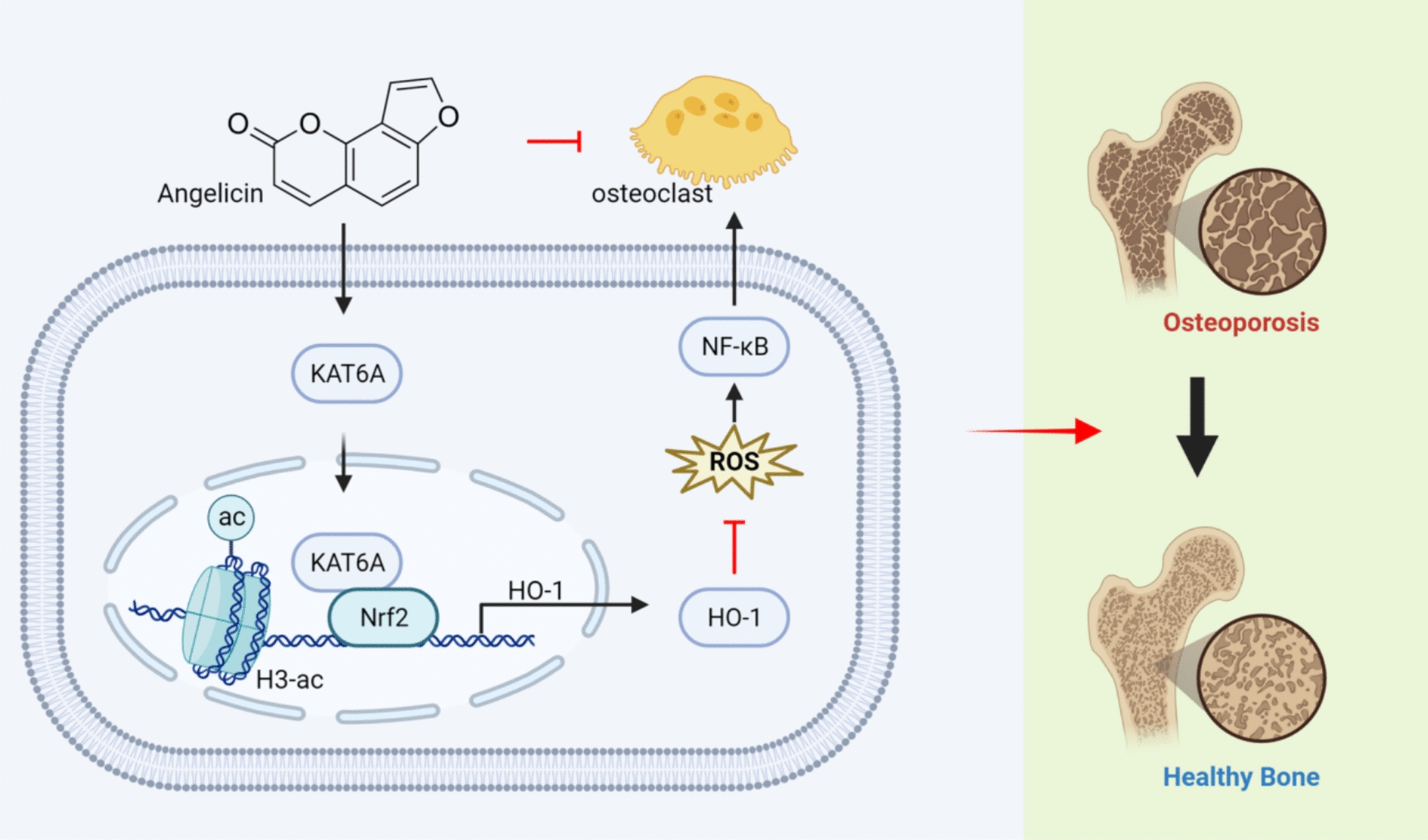
Schematic of the mechanism by which angelicin inhibits in vivo and in vitro osteoclastogenesis (Created with BioRender.com)

In summary, angelicin reduces osteoclast formation by inhibiting oxidative stress in the bone marrow microenvironment. Our study found for the first time that angelicin further activated the Nrf2/HO-1 antioxidant system and reduced the ROS level in osteoclasts by upregulating the level of KAT6A in osteoclasts, thus inhibiting oxidative stress levels and osteoclast formation (Fig. 10). Therefore, angelicin can be used as a potential candidate drug for treating osteoporosis.

### Supplementary Information

Supplementary Material 1. We examined the pathology of various organs in rats, which was used to prove the safety of angelicin in rats.

## Data Availability

The research data used to support the findings are available from the corresponding author upon request.

## Abbreviations

BMD: Bone density
BV/TV: Bone volume
HO-1: Heme oxygenase-1
KAT6A: Lysine acetyltransferase 6A
Keap1: Kelch-like ECH associated protein 1
Nrf2: Transcription factor nuclear factor erythroid factor 2 associated factor
ROS: Oxygen species
Tb. N: Trabecular number
Tb. Th: Trabecular thickness
Tb. Sp: Trabecular separation
