# Supplementary figures and images for: Angelicin improves osteoporosis in ovariectomized rats by reducing ROS production in osteoclasts through regulation of the KAT6A/Nrf2 signalling pathway
Source: Chin Med. 2024 Jul 2;19:91. doi: 10.1186/s13020-024-00961-7 (PMC11218408; doi:10.1186/s13020-024-00961-7)

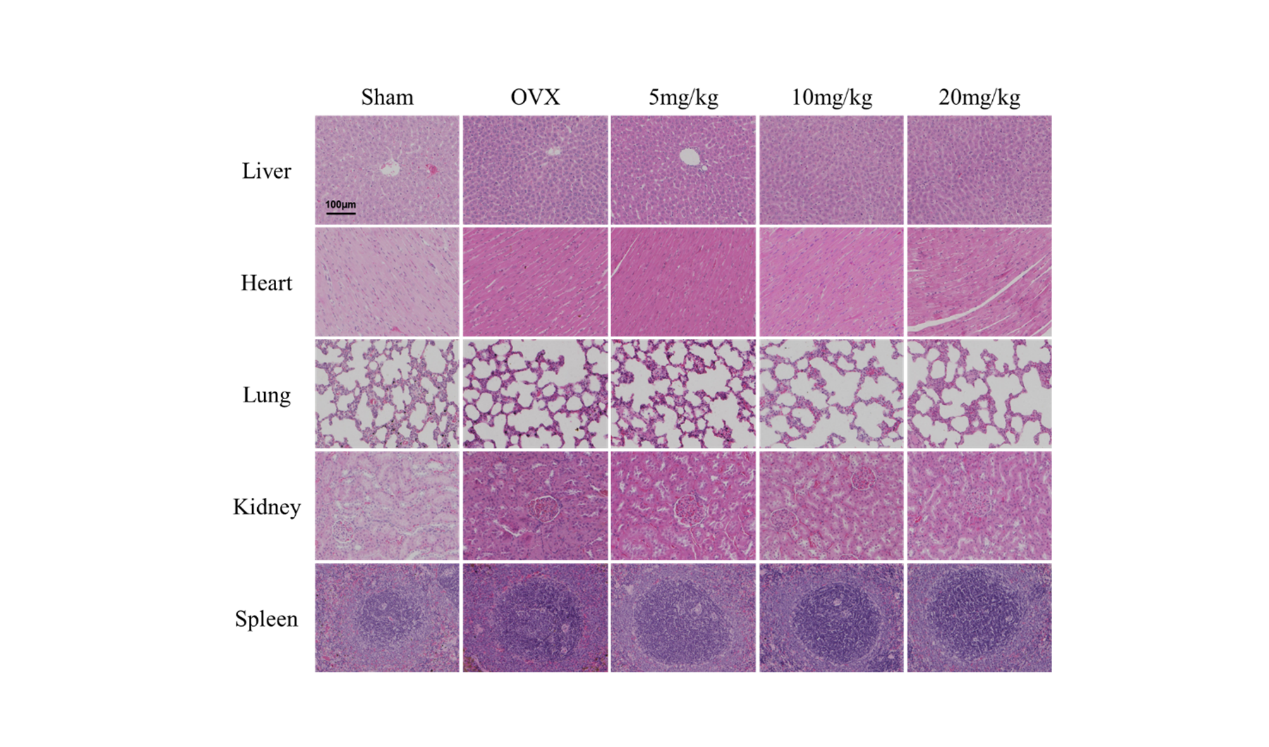


**Supplementary Figure**

Supplement: Supplementary file 1 — Supplementary Material 1. We examined the pathology of various organs in rats, which was used to prove the safety of angelicin in rats. [file 13020_2024_961_MOESM1_ESM.docx]
